# *Cyclocarya paliurus* ethanol leaf extracts protect against diabetic cardiomyopathy in db/db mice via regulating PI3K/Akt/NF-κB signaling

**DOI:** 10.29219/fnr.v64.4267

**Published:** 2020-08-31

**Authors:** Yang Wang, Xiaojie Zheng, Longyu Li, Hong Wang, Keyuan Chen, Mingjie Xu, Yiwei Wu, Xueli Huang, Meiling Zhang, Xiaoxia Ye, Tunhai Xu, Rongchang Chen, Yindi Zhu

**Affiliations:** 1 School of Pharmaceutical Sciences, Wenzhou Medical University, Wenzhou, China; 2 School of Chinese Pharmacy, Beijing University of Chinese Medicine, Beijing, China; 3 Wenzhou Vocational College of Science & Technology, Wenzhou, China; 4 State Key Laboratory of Quality Research, Chinese Medicine and School of Pharmacy, Macau University of Science and Technology, Macau, China; 5 Key Laboratory of Bioactive Substances and Resources Utilization of Chinese Herbal Medicine, Ministry of Education, Institute of Medicinal Plant Development, Peking Union Medical College and Chinese Academy of Medical Sciences, Beijing, China

**Keywords:** Cyclocarya paliurus, diabetic cardiomyopathy, inflammation, PI3K/Akt, NF-κB

## Abstract

**Background:**

Diabetic cardiomyopathy (DCM) is a serious complication of diabetes that can lead to significant mortality. *Cyclocarya paliurus* is a tree, the leaves of which are often utilized to prevent and treat diabetes mellitus. Whether *C. paliurus* leaves can prevent or treat DCM, however, it remains to be formally assessed. The present study was therefore designed to assess the ability of *C. paliurus* to protect against DCM in db/db mice.

**Methods:**

Male wild-type (WT) and db/db mice were administered *C. paliurus* ethanol leaf extracts (ECL) or appropriate vehicle controls daily via gavage, and levels of blood glucose in treated animals were assessed on a weekly basis. After a 10-week treatment, the levels of cardiac troponin I (cTn-I), lactate dehydrogenase (LDH), creatine kinase MB (CK-MB), aspartate transaminase (AST), total triglycerides (TG), and total cholesterol (TC) in serum were measured. Activities of malondialdehyde (MDA), superoxide dismutase (SOD), glutathione peroxidase (GSH-Px), and catalase (CAT) and the levels of tumor necrosis factor-α (TNF-α), interleukin-1β (IL-1β), and IL-6 in heart tissues were detected. Hematoxylin-eosin (HE) and Masson staining were conducted. The protein expression that related with oxidative stress and inflammatory reaction was evaluated by Western blotting.

**Results:**

Compared with WT mice, the TG, TC, and blood glucose levels in db/db mice increased significantly, which were reduced by ECL treatment. Compared with WT mice, the levels of LDH, CK-MB, AST, and cTn-I in serum and MDA in heart tissues of db/db mice increased significantly. Activities of SOD, GSH-Px, and CAT in heart tissues of db/db mice decreased significantly. The levels of inflammatory cytokines (TNF-α, IL-1β, and IL-6) in heart tissues of db/db mice increased remarkably. However, ECL treatment improved the above pathological changes significantly. ECL alleviated pathological injury and fibrosis in heart tissues of mice. Western blotting showed that ECL increased Bcl-2 level and decreased Bax, cle-caspase-3, and cle-caspase-9 expression. Furthermore, ECL inhibited NF-κB nuclear translocation and increased PI3K and p-Akt expressions.

**Conclusion:**

Our results indicate that ECL treatment can markedly reduce pathological cardiac damage in db/db mice through antiapoptotic, antifibrotic, and anti-inflammatory mechanisms. Specifically, this extract was able to suppress NF-κB activation via the PI3K/Akt signaling pathway. Given its diverse activities and lack of significant side effects, ECL may thus have therapeutic value for the treatment of DCM.

## Popular scientific summary

- *Cyclocarya paliurus* ethanol leaf extract (ECL) is able to protect against the development of diabetic cardiomyopathy in db/db mice.
- The protective efficacy of ECL is associated with antioxidant, antiapoptotic, and anti-inflammatory mechanisms.
- ECL is able to inhibit the activation and nuclear localization of NF-κB via promoting PI3K/Akt signaling in db/db mice.

Diabetes mellitus is an increasingly common disease that currently affects nearly 500 million people globally (1). The chronic hyperglycemia that is common in patients with diabetes patients can drive the onset of nerve damage, vision problems, heart disease, and potentially a need for amputation (2). Diabetic cardiomyopathy (DCM) affects 15–20% of diabetes patients (3) and is thought to account for between 50 and 80% of deaths in this patient population (4), making it a global health threat. DCM is characterized by cardiac fibrosis, myocardial left ventricular dysfunction, cardiac hypertrophy, and the apoptotic death of cardiomyocytes (5). DCM is thought to be driven by a number of physiological changes including metabolic alterations, mitochondrial dysfunction, impaired calcium homeostasis, increased inflammation and oxidative stress, and advanced glycation end product accumulation (6–8).

PI3K/Akt signaling is a key intracellular pathway that is essential for directing appropriate responses to extracellular stimulation, governing diverse biological processes relevant to DCM such as proliferation, apoptosis, and inflammation (9). Diabetic complications are further induced and aggravated through the actions of intracellular and extracellular proteins including NF-κβ, transforming growth factor-β (TGF-β), poly (ADP-ribose) polymerase (PARP), interleukin-6 (IL-6), interleukin-1β (IL-1β), mitogen-activated protein kinase (MAPK), and vascular endothelial growth factor (VEGF) (10). Despite its growing prevalence and many researches on DCM at present, there are few effective methods for the treatment of DCM in clinical. Therefore, it is of great significance to develop drugs for the treatment of DCM (11, 12).

In recent years, there has been a resurgence of interest in the use of natural herbs as a source for medicinal compounds, offering potentially viable novel treatments for diabetes patients (11, 12). The herbal compounds employed in traditional Chinese medicine (TCM) offer certain advantages including their multipotent therapeutic effects and their relatively low rates of toxicity or other side effects (13). *Cyclocarya paliurus* (Batalin) Iljinsk. (Juglandaceae) is one of such multifunctional species of tree which is found in forests and mountainous regions throughout southern and southeastern China (14). The leaves of this tree are commonly employed both as a tea or a food additive, and medicinal purposes in TCM applications wherein they are used to treat diabetes, hypertension, and hyperliposis (15). Previous studies of *C. paliurus* have revealed that its extracts contain triterpenoids (16), flavonoids, phenolic compounds (17), and polysaccharides (18), all of which can mediate reductions in blood glucose and lipid levels, decreasing blood pressure and insulin resistance. These previous studies, however, failed to determine whether *C. paliurus* offers any efficacy in the treatment of DCM. In this study, we therefore explored the ability of a *C. paliurus* ethanol leaf extract (ECL) to combat DCM in db/db mice. The results of this study highlight the potential utility of ECL as a means of treating or preventing DCM development.

## Materials and methods

### Plant extract preparation

*C. paliurus* leaves were obtained from Wencheng Shengshan Food Development Co. Ltd. (Wenzhou, Zhejiang, China), with Professor Hong Wang of the School of Pharmacy, Wenzhou Medical University confirming the identity of these leaves, and with a voucher specimen (No. 20180613) being deposited at this institution. A total of 35 kg of dried leaves underwent two extractions (2 h/extraction) with 350 L of 70% ethanol (v/v) at 80°C. This approach yielded 7 kg of crude extract (20% of input), and after filtration, the solvent was removed under reduced pressure.

### Chemicals and reagents

ACCU-CHEK Active blood glucose test strips were obtained from Roche (NJ, USA). Triglyceride (TG) and total cholesterol (TC) levels, as well as superoxide dismutase (SOD), glutathione peroxidase (GSH-Px), catalase (CAT), and malondialdehyde (MDA) activities, were measured using kits from Jiancheng Bioengineering Institute (Nanjing, China). Enzyme-linked immunosorbent assay (ELISA) kits specific for collagen I, cardiac troponin I (cTn-I), tumor necrosis factor-α (TNF-α), IL-1β, and IL-6 were obtained from HaiTai TongDa Sci Tech Ltd (Beijing, China). Antibodies specific for caspase-3, caspase-9, Bcl-2, Bax, NFκB, AKT, p-AKT, β-actin, and lamin B were obtained from Abcam (MA, USA). horseradish peroxidase (HRP)-conjugated secondary antibodies were obtained from CW Biotech (Beijing, China). All other chemicals were obtained from Sigma (St. Louis, MO, USA) and were of analytical grade or higher.

### Animal model development

A total of 10 male C57BL/6J and 40 male C57BL/KsJ db/db mice (all 20 ± 2 g; 8 weeks old) were obtained from Shanghai Slac Laboratory Animal Co. Ltd. (Shanghai, China). Mice were housed in a facility maintained at 24°C ± 1°C with 50 ± 5% humidity and a 12 h light/dark cycle. Animals had free access to food and water and were treated in a manner consistent with the Guide for the Care and Use of Laboratory Animals (NIH, USA). The Wenzhou Medical University Animal Care and Use Committee approved all studies described herein.

Mice were allowed to acclimate for 1 week after acquisition. Wild-type (WT) mice were then allocated to the (1) C57BL/6 group (control), while db/db mice were randomized into four groups (*n* = 10/group), (2) db/db (model); (3) db/db + ECL 20 mg/kg/day (low); (4) db/db + ECL 40 mg/kg/day (intermediate); and (5) db/db + ECL 80 mg/kg/day (high). Control and model group animals were administered purified water in lieu of ECL. Water and ECL were administered to appropriate groups of mice via oral gavage once per day for 5 days/week over a 10-week period.

### Glucose measurement and sample collection

Once per week, mice were subjected to the measurement of postprandial blood glucose levels. Briefly, food was removed from animals for 2 h, after which tail vein blood samples were collected and a glucose monitor was used to quantify glucose levels therein. At the end of the treatment period, animals were intraperitoneally injected with 50 mg/kg pentobarbital for euthanasia. Aortic blood samples were then collected and spun for 10 min at 3,500 rpm at 4°C, 3,500 rpm for 10 min. Hearts were also collected from all mice and were rinsed in cold saline solution (0.9%) prior to careful removal of the vessels, connective tissue, atrial tissue, and right ventricular tissue. Samples were then split into half, with one half being frozen for Western blotting and the other being fixed using 10% neutral buffered formalin prior to pathological examination.

### TG and TC measurements

Both TG and TC levels in murine serum were measured with commercial kits (Nanjing Jiancheng Bioengineering, Nanjing, China) based on the provided directions. All measurements were collected during the same time period to ensure consistency.

### Cardiac biomarker measurements

Serum lactate dehydrogenase (LDH), creatine kinase MB (CK-MB), and aspartate transaminase (AST) activities were measured using commercial kits (Nanjing Jiancheng Bioengineering, Nanjing, China) based on the provided directions. In addition, serum cTn-I levels were measured via ELISA.

### Cardiac oxidative stress analysis

Cardiac tissue was combined with saline solution at a ratio of 1:9 mg/µL to yield a homogenate that was then spun for 5 min at 7,000 rpm, after which supernatants were collected and used for SOD, GSH-Px, CAT, and MDA measurements based on the provided kit directions.

### Inflammatory cytokine measurement

Heart tissues were mixed with saline at a ratio of 1:9 mg/µL to form a homogenate. After centrifugation at 7,000 rpm for 5 min, the supernatant was used to measure the levels of IL-1β, TNF-α, and IL-6 by ELISA according to the manufacturer’s instructions (Hai Tai Tong Da Sci Tech Ltd, Beijing, China).

### Pathological staining

Cardiac tissue samples that were fixed as above were dehydrated and paraffin-embedded, after which they were cut into 5 μm sections that were either hematoxylin and eosin (H&E) stained or subjected to Masson’s trichrome staining in order to visualize extracellular matrix deposition. A light microscope (CKX41, 170 Olympus, Tokyo, Japan) was then used to assess all stained tissue sections, with a pathologist that was blinded to group assignments conducting all analyses thereof.

### Western blotting

Total protein was extracted from cardiac tissue samples, and protein levels in these extracts were quantified via bicinchoninic acid (BCA) assay. Samples were then separated via 12% sodium dodecyl sulfate-polyacrylamide gel electrophoresis (SDS-PAGE) prior to transfer onto nitrocellulose membranes. The resultant blots were blocked for 2 h with 5% non-fat milk in tris-buffered saline with Tween 20 (TBST), followed by overnight incubation at 4°C with appropriate primary antibodies. Blots were then washed thrice with TBST prior to staining with appropriate secondary antibodies for 2 h. A GelDox XR System (Bio-Rad, CA, USA) and the Quantity One software were used for protein band detection.

### Statistical analysis

SPSS v16.0 and GraphPad Prism 8 (GraphPad, CA, USA) were employed for all statistical testing. Data are means ± standard deviations from triplicate experiments. Data were compared via one-way ANOVAs followed by a multiple comparison test with Bonferroni’s correction. *P* < 0.05 was the significance threshold.

## Results

### ECL treatment alters blood glucose, TG, and TC levels in db/db mice

**Fig. 1:**
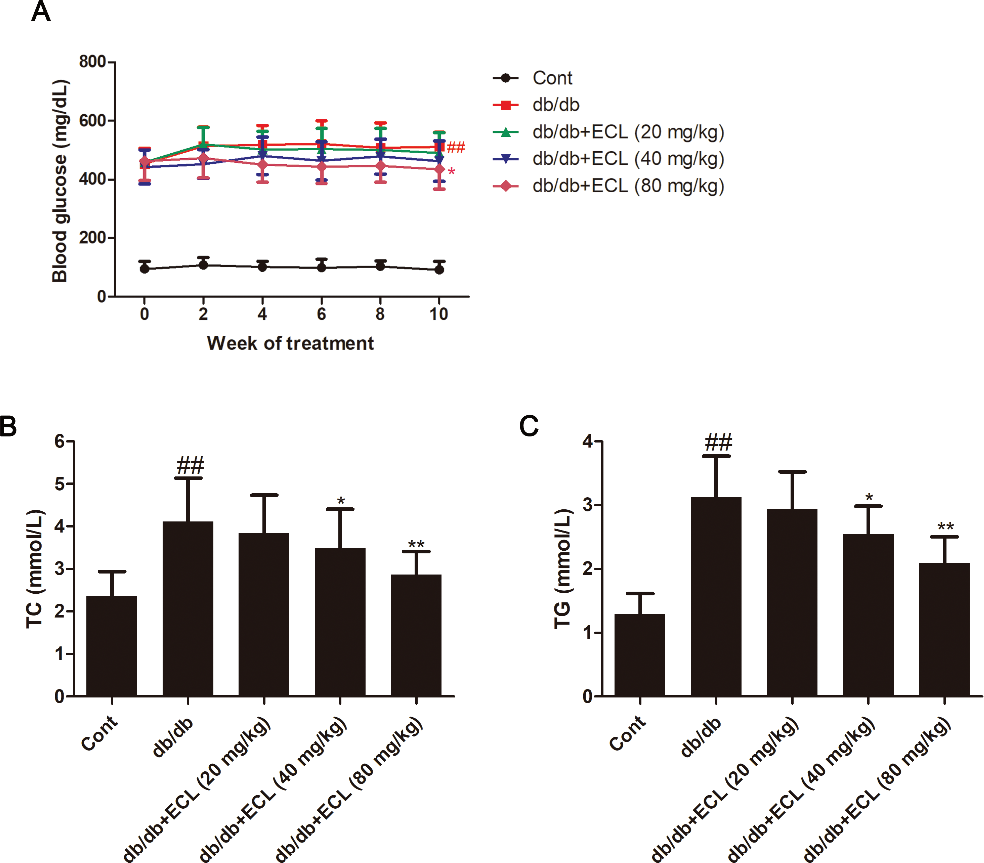
ECL treatment alters serum lipid levels in db/db mice. Serum (A) blood glucose, (B) TC, and (C) TG levels were measured. ^##^*P* < 0.01 versus control group; *P < 0.05, **P < 0.01 versus db/db group. Data are means ± standard deviations.

In the present study, db/db mice were used because they are a frequently utilized animal model of type 2 diabetes mellitus (T2DM), in which the mechanistic basis for DCM can be reliably studied (19). In order to monitor the impact of ECL in treated animals, postprandial blood glucose levels were quantified in the five different mouse groups over the 10-week treatment period. This revealed that db/db model mice exhibited significantly higher blood glucose levels than vehicle-treated WT controls (Fig. 1A). This elevated blood glucose declined in ECL-treated animals, with decreases being evident within 2 weeks in animals administered a high (80 mg/kg) dose. After 10 weeks, mice in the 40 and 80 mg/kg/day ECL groups exhibited 12.4 and 17.6% reductions in blood glucose levels, respectively, relative to db/db model mice. Elevated levels of TG and TC are closely linked to hyperlipidemia (20), making reductions in these levels a goal of effective hypolipidemic interventions. We therefore measured TG and TC levels in treated animals after 10 weeks, revealing that db/db model animals exhibited significant increases in serum TG and TC relative to WT controls. Importantly, treatment with 40 and 80 mg/kg ECL was sufficient to ameliorate these elevated serum lipid levels (Fig. 1B–C). These results therefore suggested that ECL treatment in db/db mice can alter metabolic pathways so as to induce a hypolipidemic effect.

### ECL treatment alters cardiac biomarker activity

**Fig. 2:**
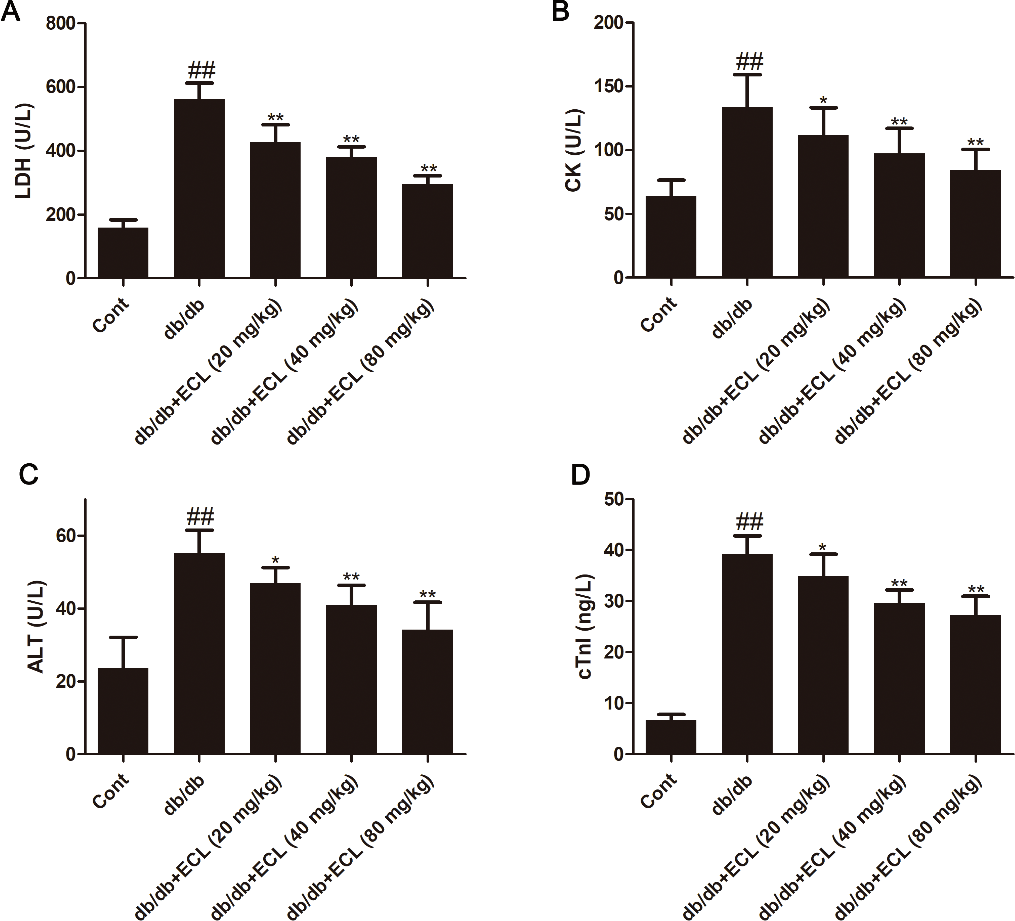
ECL alters serum myocardial enzyme activity and cTnI levels. Serum LDH (A), CK (B), AST (C), and cTnI (D) was quantified using appropriate assay kits. ^##^ *P* < 0.01 versus control group; *P < 0.05, **P < 0.01 versus db/db group. Data are means ± standard deviations.

A number of different markers including LDH, CK-MB, AST, and cTn-I are considered to be characteristic of cardiomyocytes, with their release into serum suggesting the death or dysfunction of these cardiac cells. DCM has previously been shown to coincide with increased serum cTn-I levels and elevated serum LDH, CK-MB, and AST activity (21). Consistent with this, we observed increased cTn-I levels and CK-MB, LDH, and AST activity in the serum of db/db model animals relative to WT controls, whereas ECL treatment significantly reversed these effects (*P* < 0.05 or *P* < 0.01, Fig. 2).

### ECL suppresses oxidative stress in cardiac tissue

**Fig. 3:**
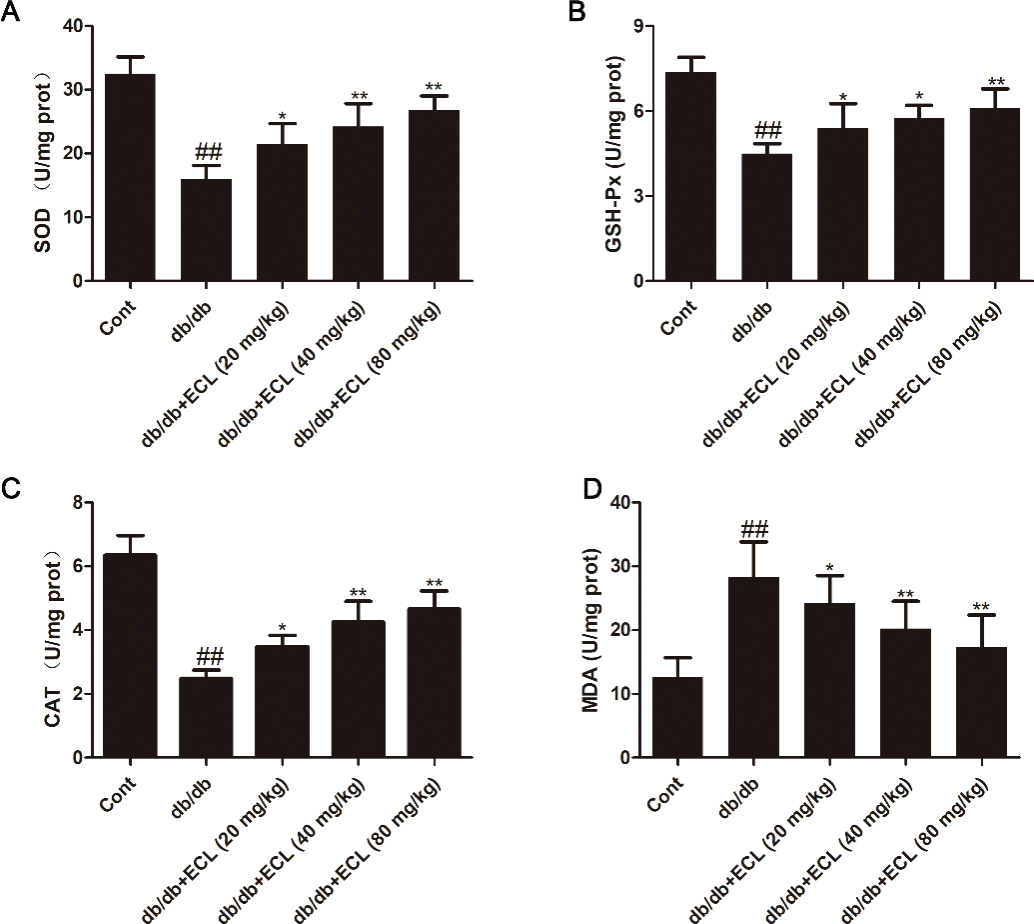
ECL suppresses cardiac oxidative stress in db/db mice. SOD (A), GSH-Px (E), CAT (C), and MDA (D) activities were quantified with commercial assay kits. ^##^ *P* < 0.01 versus control group; *P < 0.05, **P < 0.01 versus db/db group. Data are means ± standard deviations.

Chronic hyperglycemia can lead to the sustained generation of reactive oxygen species (ROS) capable of damaging macromolecules within cells. The antioxidant enzymes SOD, CAT, and GSH-Px are essential means of preventing the resultant oxidative stress and associated damage (22). The activities of these enzymes, however, are typically disrupted in the context of diabetes (23), with significantly increased SOD, GSH-Px, and CAT activity and significantly reduced MDA activity being evident in the cardiac tissue of diabetic mice relative to healthy WT controls. We observed these changes in our model system and found that a 10-week treatment with ECL was sufficient to reverse these changes (Fig. 3), indicating that ECL can inhibit cardiac oxidative stress in db/db mice.

### ECL treatment alters myocardial tissue pathology in db/db mice

**Fig. 4:**
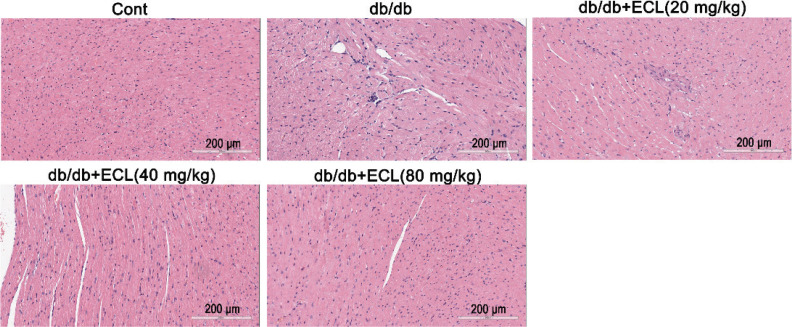
ECL alleviates hypertrophic cardiac injury in db/db mice. H&E staining of cardiac tissue from WT control, db/db, and ECL treatment groups. All images are longitudinal sections.

We next assessed the cardiac tissue of mice in our different treatment groups for evidence of morphological changes or functional injury. We found that WT control mice exhibited normally and tightly arranged myocardial cells with a defined structure and relatively limited extracellular matrix deposition, whereas db/db model mice showed evidence of hypertrophic growth, distorted myocardial cell arrangements, and focal inflammatory cell infiltration. In contrast, ECL treatment of db/db mice was associated with the amelioration of these pathological changes in cardiac morphology, suggesting that low, intermediate, and high (20, 40, or 80 mg/kg, respectively) ECL doses were sufficient to alleviate DCM-associated cardiomyocyte hypertrophy in these model animals (Fig. 4).

### ECL ameliorates myocardial fibrosis

**Fig. 5:**
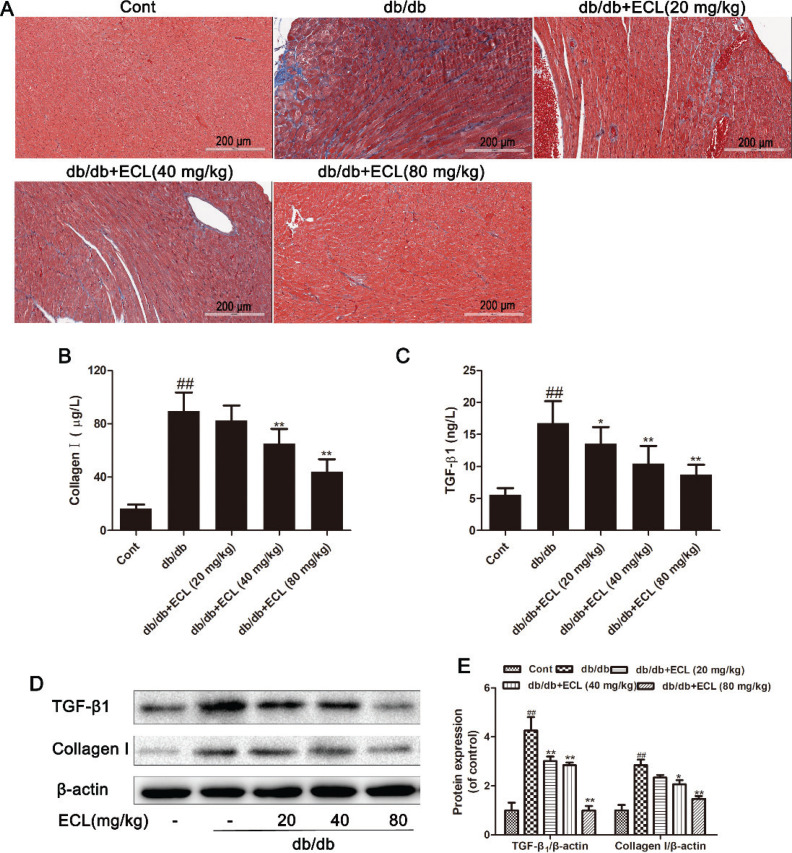
ECL suppresses cardiac fibrosis in db/db mice. (A) Masson staining of cardiac tissue. (B, C) Cardiac collagen I and TGF-β1 levels were measured via ELISA. (D) Cardiac collagen I and TGF-β1 levels were measured via Western blotting. (E) Relative collagen I and TGF-β1 expression was quantified. ^##^ *P* < 0.01 versus control group; *P < 0.05, **P < 0.01 versus db/db group. Data are means ± standard deviations.

We next used Masson’s trichrome staining and other methods to assess the incidence of cardiac fibrosis in our mice. We found that db/db model group animals exhibited clear evidence of fibrosis, with a disorganized myocardial, perivascular, and interstitial collagen network structure being evident. Treatment with all tested doses of ECL (20, 40, or 80 mg/kg) was able to alleviate these fibrotic changes in the cardiac tissue of treated animals in a dose-dependent manner. In addition, ECL significantly reduced the expression of key indicators of myocardial fibrosis including collagen I (Fig. 5B) and TGF-β1 (Fig. 5C), as assessed via ELISA and Western blotting (Fig. 5D). This thus suggests that ECL treatment can alleviate cardiac fibrosis in db/db mice.

### ECL reduces myocardial inflammation

**Fig. 6:**
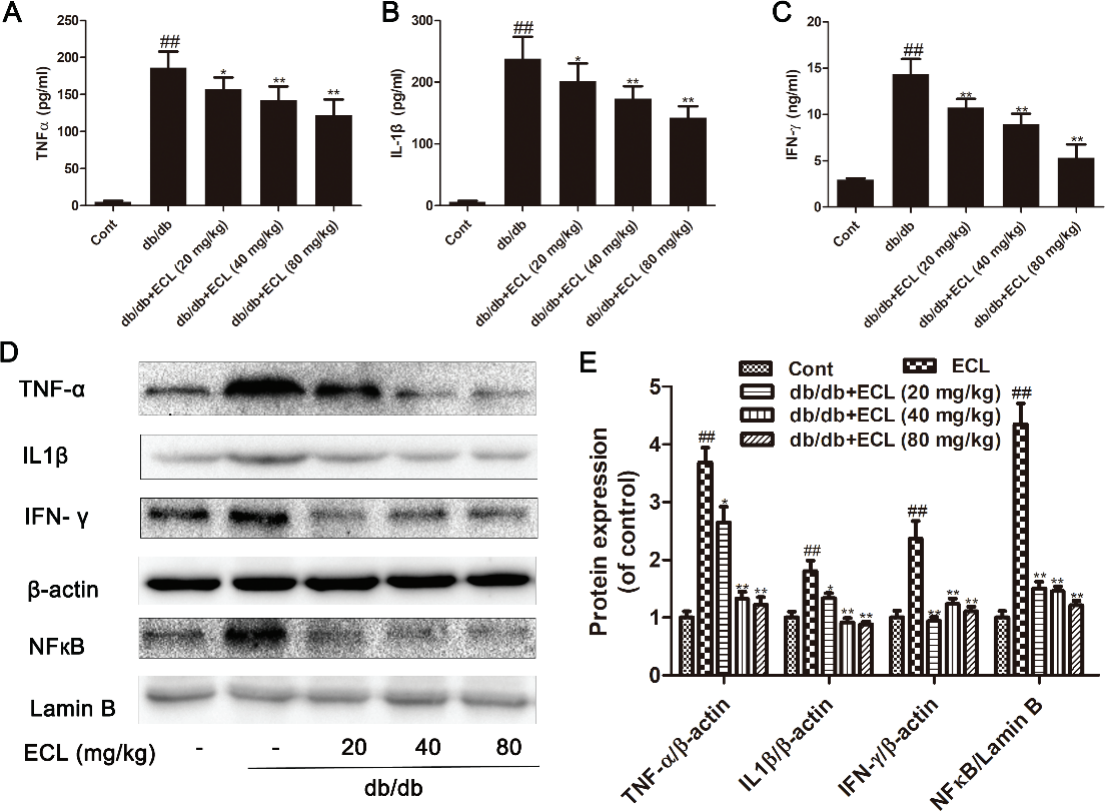
ECL suppresses inflammation in db/db mice. Cardiac TNF-α (A), IL-1β (B), and IL-6 (C) levels were measured via ELISA. (D) Cardiac TNF-α, IL-1β, IFN-γ, and NF-κB levels were measured via Western blotting. (E) Quantification of protein expression. ^##^ *P* < 0.01 versus control group; *P < 0.05, **P < 0.01 versus db/db group. Data are means ± standard deviations.

We next assessed inflammatory cytokine levels in the myocardial tissues of mice in the present study, revealing that levels of TNF-α, IL-1β, and IL-6 were significantly elevated in the cardiac tissue of db/db mice related to WT controls as confirmed via ELISA and Western blotting (Fig. 6D and E). Treatment for 10 weeks with 40 or 80 mg/kg ECL was sufficient to mediate a dose-dependent reduction in the levels of all three of these inflammatory cytokines (*P* < 0.05 or *P* < 0.01) (Fig. 6A–C). Furthermore, we found that NF-κB expression was significantly lower in ECL-treated animals related to diabetic controls, thus suggesting that ECL is able to inhibit inflammation in diabetic mice via promoting NF-κB downregulation.

### ECL treatment alters apoptotic protein expression

**Fig. 7:**
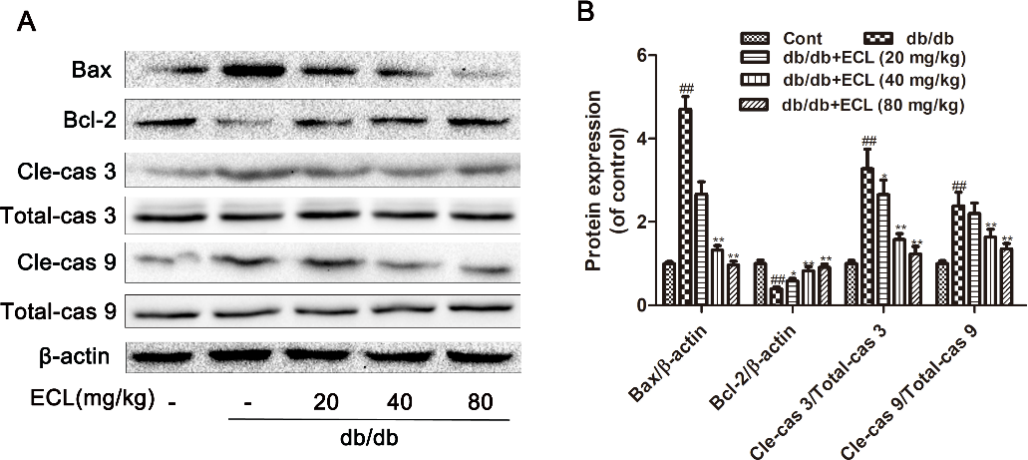
ECL suppresses myocardial apoptosis. (A) Cardiac cle-caspase-3, total-caspase-3, cle-caspase-9, total-caspase-9, Bax, and Bcl-2 levels were measured via Western blotting. (B) Quantification of protein expression, *n* = 3. ^##^ *P* < 0.01 versus control group; *P < 0.05, **P < 0.01 versus db/db group. Data are means ± standard deviations.

We next used Western blotting to assess the expression of apoptosis-related cle-caspase-3, total-caspase-3, cle-caspase-9, total-caspase-9, Bax, and Bcl-2 proteins in the cardiac tissue of our model mice. Relative to WT controls, db/db model mice exhibited reduced antiapoptotic Bcl-2 expression and increased proapoptotic cle-caspase-3, cle-caspase-9, and Bax expression (*P* < 0.05 or *P* < 0.01). ECL treatment of these animals was, in contrast, associated with significant increases in Bcl-2 expression and with significant reductions in Bax, cle-caspase-3, and cle-caspase-9 levels (Fig. 7). These results suggest that ECL is able to suppress the diabetes-induced apoptotic death of cardiomyocytes that occur in db/db mice.

### ECL impacts the PI3K/Akt signaling pathway

**Fig. 8:**
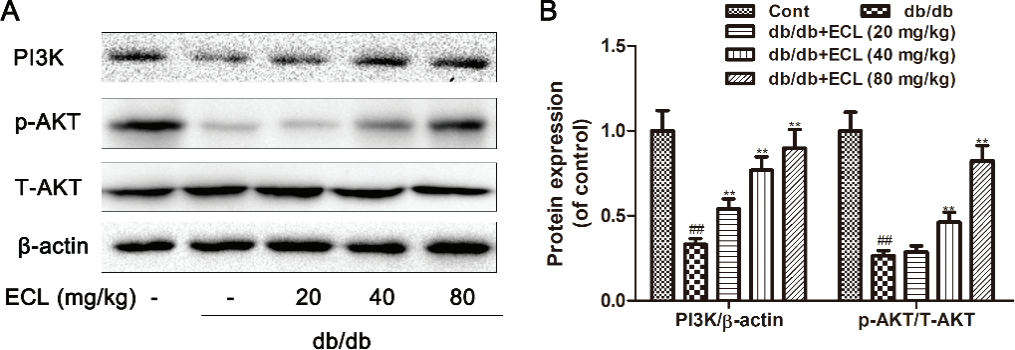
ECL treatment enhances cardiac PI3K/Akt signaling in diabetic mice. (A) Cardiac PI3K and p-Akt levels were measured via Western blotting. (B) Quantification of protein expression. ^##^ *P* < 0.01 vs. control group; *P < 0.05, **P < 0.01 versus db/db group. Data are means ± standard deviations.

PI3K/Akt signaling is essential for cellular responses to extracellular stimuli, and it can effectively regulate inflammatory responses. To explore whether ECL mediates its anti-inflammatory activity via the PI3K pathway, we analyzed the impact of ECL treatment on PI3K and Akt phosphorylation in cardiac tissue samples by Western blotting. Relative to WT controls, we observed significant reductions in PI3K and Akt phosphorylation in db/db model animals, whereas oral ECL treatment reversed this change in a dose-dependent manner (Fig. 8A and B).

## Discussion

Diabetes is a highly prevalent disease that currently affects 8.8% of the global population, with this incidence expected to rise to 10.4% by 2040 (24). The development of DCM in T2DM patients is associated with serious complications including cardiac fibrosis, hypertrophy, and potentially heart failure (25). Many recent studies have explored the potential application of TCM herbs as medicines for the treatment of T2DM, with many efficacious natural extracts having been prepared to combat this disease and its complications (26). *C. paliurus* is a medicinal plant that has long been used as a tea and dietary supplement for medicinal purposes in the area in which it grows naturally. Furthermore, in 1999 a *C. paliurus*-derived tea became the first FDA-approved health tea in China (27). Indeed, there is a growing evidence suggesting that *C. paliurus* leaves possess a range of pharmacological activities including the ability to reduce hypertension, hyperglycemia, and hyperlipidemia (28).

In this study, we provided a novel evidence that ECL treatment can protect against structural and functional changes in the cardiac tissue of diabetic mice. This cardioprotective activity was attributable to the antioxidant, antiapoptotic, anti-inflammatory, and antifibrotic activity of ECL. Oral administration of ECL to db/db mice was sufficient to prevent the development of cardiac hypertrophy, myocardial fibrosis, and associated oxidative stress and inflammation in treated animals. ECL further lowered blood lipid levels in treated animals. Together, these findings indicate that ECL has value as a therapeutic compound capable of treating and preventing DCM.

While the specific molecular basis for DCM remains to be fully clarified, it is thought to develop in part through an inflammatory mechanism (29, 30). The hyperglycemic state that exists in T2DM patients can drive the increased production of intracellular ROS, thereby driving tissue injury and inflammation (31, 32). Inflammatory cardiomyocyte signaling is associated with the overproduction of both cytosolic and mitochondrial ROS (33, 34). Hyperglycemia is indeed a key known driver of oxidative stress and inflammation, thereby driving cardiac fibrosis and reduced cardiac function (35). Such myocardial inflammation can drive DCM development with NF-κB signaling within the cardiac tissue being a major driver of this disease progression (36). There are five different NF-κB family proteins that can interact to form homo- and heterodimeric complexes (37). At baseline, NF-κB is sequestered in the cytosol by IκB proteins. However, in response to stimuli including intracellular oxidative stress, IκBs are phosphorylated and degraded, thus allowing NF-κB to undergo nuclear translocation and to mediate the subsequent upregulation of inflammatory cytokines (TNF, Il-6, IL-8) (38). Such NF-κB is thought to be a central driver of DCM progression (39). NF-κB is potentially involved in all major cardiac responses to diabetes including inflammation, fibrosis, hypertrophy, and apoptosis (40). We found that ECL was able to reduce pathological injury in the cardiac tissue of db/db mice through a mechanism associated with the antioxidant, antiapoptotic, anti-inflammatory, and antifibrotic activity of this extract, which also decreased NF-κB expression. As such, *C. paliurus* extracts may be ideal for treating or preventing DCM.

**Fig. 9:**
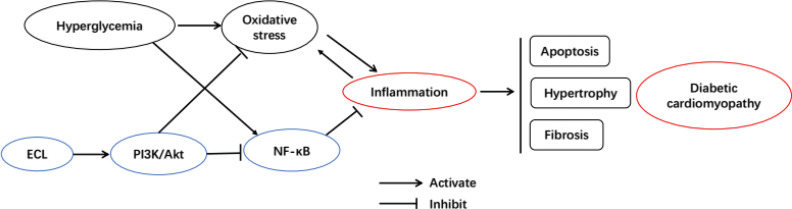
ECL alleviates cardiac inflammation in db/db mice via PI3K/Akt pathway activation and inhibition of NF-κB signaling.

PI3K family kinases are important generators of lipid second messenger molecules that can recruit cytoplasmic proteins to the cellular membrane. Akt is a downstream target of PI3K activation, regulating anabolic processes via phosphorylating roughly 300 different substrates when it is activated in cells (41). PI3K/Akt signaling is essential for the regulation of inflammatory responses, at least in part via controlling intracellular NF-κB activity (42). Indeed, PI3K and Akt can drive NF-κB activation in response to IL-1 and TNF-α (43). We found that ECL treatment resulted in dose-dependent increases in PI3K expression and Akt phosphorylation, suggesting that the observed reductions in NF-κB expression in treated mice may be attributable to ECL-induced changes in PI3K/Akt pathway activity (Fig. 9).

## Conclusions

In summary, the results of this study indicate that ECL treatment can induce a significant reduction in blood glucose levels and can alleviate pathological cardiac tissue damage in db/db mice. This suggests that ECL may represent a viable treatment or dietary supplement for individuals suffering from T2DM due to its cardioprotective effects. We found that ECL is able to prevent cardiac injury via reducing inflammation, oxidative stress, apoptosis, and metabolic changes in treated animals. The mechanistic basis for these activities seems to be related to reductions in NF-κB expression via PI3K/Akt signaling pathway activation.
